# Critical Parameters for Efficient Sonication and Improved Chromatin Immunoprecipitation of High Molecular Weight Proteins

**DOI:** 10.1371/journal.pone.0148023

**Published:** 2016-01-28

**Authors:** Nikolay A. Pchelintsev, Peter D. Adams, David M. Nelson

**Affiliations:** CR-UK Beatson Labs, Institute of Cancer Sciences, University of Glasgow, Glasgow, United Kingdom; University of North Dakota, UNITED STATES

## Abstract

Solubilization of cross-linked cells followed by chromatin shearing is essential for successful chromatin immunoprecipitation (ChIP). However, this task, typically accomplished by ultrasound treatment, may often become a pitfall of the process, due to inconsistent results obtained between different experiments under seemingly identical conditions. To address this issue we systematically studied ultrasound-mediated cell lysis and chromatin shearing, identified critical parameters of the process and formulated a generic strategy for rational optimization of ultrasound treatment. We also demonstrated that whereas ultrasound treatment required to shear chromatin to within a range of 100–400 bp typically degrades large proteins, a combination of brief sonication and benzonase digestion allows for the generation of similarly sized chromatin fragments while preserving the integrity of associated proteins. This approach should drastically improve ChIP efficiency for this class of proteins.

## Introduction

Chromatin immunoprecipitation (ChIP) is a powerful method to study DNA-protein interactions *in vivo* and is widely used in the fields of chromatin biology and transcription regulation [1–4]. When studying transcription factors and other proteins that only transiently associate with DNA, these DNA-protein interactions have to be stabilized by covalent cross-linking [5]. This is usually achieved by formaldehyde treatment, although alternative cross-linking agents have also been described [6,7]. Cross-linked cells become resistant to detergent-mediated lysis and more powerful methods must be implemented in order to efficiently solubilize this material. Ultrasound treatment [8,9] is one of the most popular solutions because it combines cell lysis and non-specific DNA shearing, generating small chromatin fragments that are well suited for immunoprecipitation and accurate mapping of protein binding sites. In biological laboratories, sonication is frequently performed in water bath sonicators such as the Bioruptor (Diagenode), as they minimize the risk of cross contamination between samples and have a number of other advantages. However, we and others have observed that seemingly identical sonication conditions often yield inconsistent chromatin fragmentation across experiments, which can translate into poor experimental reproducibility. Additionally, while characterizing the genome-wide binding profile of the histone chaperone protein HIRA [10] we noticed a drastic decrease in the amount of HIRA detected by Western blot between samples before and after sonication. To address these issues we decided to systematically study the ultrasound treatment of cross-linked cells. Consequently, we have identified critical parameters of the process and formulated a generic strategy for rational optimization of ultrasound treatment.

## Materials and Methods

### Culturing and Cross-Linking of Cells

HeLa cells were grown in DMEM (Life Technologies) supplemented with 10% fetal bovine serum, 2 mM L-glutamine, 25 U/mL penicillin, 25 μg/mL streptomycin and passaged every 3–4 days. Cells of 80–95% confluence were harvested by trypsinization, subjected to centrifugation at 300 x g and cross-linked by resuspending the pellet in 1% formaldehyde (Sigma, 252549) in PBS. Typically, 37 mL of the mixture were used per 15×10^7^ cells. Cross-linking was allowed to proceed for 15 min at room temperature (rotating wheel, 21 rpm) and stopped by addition of 1/10 the volume of 1.25 M glycine to the reaction. After an additional 5 min incubation at room temperature (rotating wheel, 21 rpm) cross-linked cells were collected by centrifugation at 300 x g for 2 min, washed with PBS and the cell pellets were frozen at -70°C.

### Extraction and Sonication

Approximately 20×10^6^ cross-linked cells were resuspended in 1 mL of extraction and lysis buffer, ELB (50 mM Tris pH 8.0, 50 mM NaCl, 0.5% SDS, 1 mM EDTA pH 8.0), supplemented with 10 μL of protease inhibitor cocktail (Sigma, P8340). The suspension was incubated for 15 min on a rotating wheel at 21 rpm (room temperature). Cells were recovered by 2 min centrifugation at 300 g and again resuspended in ELB/inhibitors for the second round of extraction. After 30 min of incubation on a rotating wheel at 21 rpm (room temperature) samples were centrifuged for 2 min at 500 x g, the supernatant was discarded and the pellet was used for sonication.

Sonication conditions varied and are specified in text. Under the optimized protocol, one pellet volume of water was added to the cell pellet and the resulting mixture was thoroughly resuspended. The total volume of the suspension was measured by pipetting, allowing calculation of the exact pellet volume by subtracting the volume of water added. Next, more water was added to achieve a 1:4 dilution of ELB (which constituted most of the pellet volume). If required, more ELB and water in a 1:4 proportion could be added to the suspension to obtain a final density of approximately 15×10^6^ cells per mL. For example, 20×10^6^ of extracted cells typically yielded 250 μL of pellet that was first resuspended in 250 μL of water, measured for total volume and combined with another 750 μL of water, resulting in a final composition of 250 μL ELB and 1000 μL of water (1:4 ratio). The mixture was then distributed as 500 μL aliquots into 1.5 mL Eppendorf tubes with a portion retained as a pre-sonication control.

**Fig 1:**
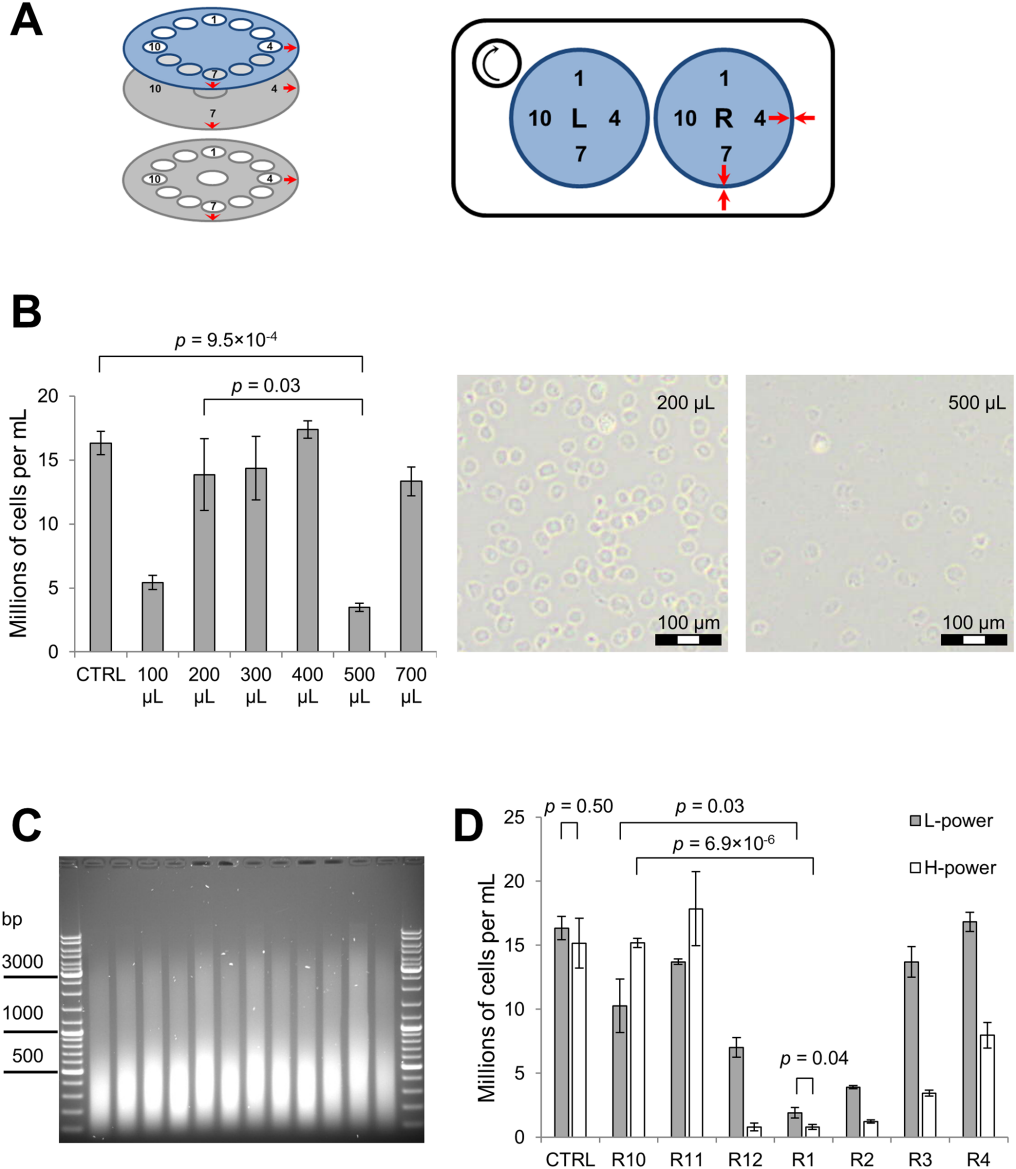
Systematic optimization of sonication conditions. **Part 1. (A)** Schematic of the Bioruptor XL water bath with tube positions numbered from 1 to 12 in the left (L) and right (R) carousels; red arrows indicate the alignment marks for carousel assembly and positioning. **(B)** The effect of sample volume on sonication efficiency at low power setting. Cell suspensions of variable volume (100–700 μL, as indicated) were loaded into positions L3, L4, L5, L9, L10 and L11. Vacant L-positions were filled with tubes containing 500 μL of water. Sonication was carried out for 8 min in 1:4 ELB:H_2_O (0.1% SDS final concentration), 24 sec ON/24 sec OFF pulses, with rotation and no floating ice. Remaining intact cells were counted three times and the respective means calculated; error bars reflect the standard deviation. The control (CTRL) sample is the cell suspension before sonication. *p*-value for analysis of variance between sonicated samples is 1.0×10^−5^, between all samples 1.4×10^−6^. *p*-values for selected T-tests are shown on the graph. **(C)** Reproducibility of sample sonication across positions L1–L12. Sonication was carried out for 40 min in 1:4 ELB:H_2_O (0.1% SDS final concentration), 24 sec ON/24 sec OFF pulses, with rotation and no floating ice. Samples were reverse cross-linked overnight and the resulting DNA was purified using the Qiagen PCR clean-up kit followed by 1.1% agarose gel analysis. **(D)** The effect of sample position and power setting on sonication efficiency. 500 μL cell suspensions were loaded into positions R10-R4 and vacant R-positions were filled with tubes containing 500 μL of water. Sonication was carried out for 1 min in 1:4 ELB:H_2_O (0.1% SDS final concentration), 5 sec ON/5 sec OFF pulses, no rotation, no ice. Intact remaining cells were counted three times and the respective means calculated; error bars reflect the standard deviation. The control (CTRL) sample is the cell suspension before sonication. *p*-value for analysis of variance between sonicated samples is 3.6×10^−9^ (L-power) and 8.2×10^−10^ (H-power). *p*-values for selected T-tests are shown on the graph.

Sonication was performed on the Diagenode Bioruptor XL instrument using the following settings: low (L) power output, 5 sec ON/5 sec OFF pulses, +4°C water bath but no floating ice, position R1 without rotation (Fig 1A, see text for more details). Total sonication time was adjusted depending on the required chromatin fragmentation: above 10,000 bp after 2 min treatment, 200–8000 bp after 4 min, 100–600 bp after 10 min and 100–400 bp after 20 min.

### Benzonase Digestion

Cells were extracted and reconstituted in ELB-water mixture (1:4) as described above. 500 μL of sample were sonicated for 2 min using the following settings: low (L) power output, 5 sec ON/5 sec OFF pulses, ice-cold water bath but no floating ice, position R1 without rotation. The sample was then brought to room temperature and 300 μL of the sample was combined with 30 μL of 10x TBS (500 mM Tris pH 8.0, 1500 mM NaCl) and 30 μL of 10% Triton X-100. Next, 3.5 μL of 100 mM MgCl_2_ was added to the mixture to achieve a 1 mM MgCl_2_ final concentration. The resulting mixture was aliquotted at 50 μL per tube (equivalent to approximately 2 μg of purified DNA per tube) and each aliquot was combined with benzonase (Sigma, E1014) titrated between 90.0 U and 0.2 U; 1 aliquot was left as a no-benzonase control. Following 15 min of benzonase digestion at room temperature, 10 μL of 0.5 M EDTA was added per tube to inactivate the benzonase. 30 μL of each aliquot was processed for overnight reverse cross-linking and DNA purification as described below (see section 2.5). The other 30 μL of each aliquot was combined with 3 μL of 1 M DTT, 12 μL of 4x Laemmli sample buffer (240 mM Tris pH 6.8, 8% SDS, 40% glycerol, 50 mM EDTA, 0.04% bromophenol blue) and incubated 30 min at +97°C to reverse the cross-links and prepare samples for Western blotting.

### Cell Counting and Statistical Analysis

10–20 μL of cell suspension were diluted 3–4 times in ELB, and 10 μL of the resulting mixture were loaded into disposable counting chambers for the Countess Automated Cell Counter (Invitrogen). Cells were manually counted on the microscope images across three different fields: at the left most part of the chamber, in the middle and in the right most part of the chamber. Average cell concentration and standard deviation (SD) were calculated based on these three readings and the size of the field studied. Pair-wise comparison between samples was performed using two-sample t-test assuming unequal variances and two-tailed distribution. Analysis of variance (ANOVA) was used to evaluate statistical significance of altering sonication conditions when multiple samples were compared within a group.

As cell counting was used to evaluate sonication efficiency we also counted large, clearly visible cell fragments together with intact cells. The rationale for this is that after successful sample sonication, all cells and fragments are completely lysed and therefore large cellular debris is indicative of incomplete sonication.

### Analysis of DNA Fragmentation

10–30 μL of the sonicated chromatin samples were combined with 10 μL of 10 mg/mL Proteinase K (Sigma, P2308), 50 μL of 2x reverse cross-linking master mix (100 mM Tris pH 8.0, 600 mM NaCl, 1.0% SDS, 100 mM EDTA pH 8.0; kept at +37°C to avoid SDS precipitation) and brought to a final volume of 100 μL with water. Two reverse cross-linking procedures were used. In the standard procedure, samples were first incubated overnight at +60°C, combined with 10 μL of 1 mg/mL RNaseA (Qiagen, 1018048) the following morning and incubated for an additional 1 h at +60°C. The resulting DNA was purified with a PCR purification kit (Qiagen, 28104). In the second procedure, referred to as the “quick” procedure, samples were incubated for 1 h at +60°C, and the resulting DNA was purified with a PCR purification kit (Qiagen, 28104). The traditional overnight procedure was used in most cases, except for two samples that were prepared using the quick procedure, as indicated in text. The advantages of each procedure are discussed in the next section. Purified DNA was analysed on ethidium bromide stained agarose gels.

### Protein Analysis

Samples were combined with 4x reducing Laemmli sample buffer (240 mM Tris pH 6.8, 8% SDS, 40% glycerol, 50 mM EDTA, 1 M DTT, 0.04% bromophenol blue), incubated 30 min at +97°C to reverse the cross-links and resolved by SDS-PAGE using 4–17% gradient gels. After that gels were either stained with Coomasie Blue or transferred to PVDF membranes and probed with protein-specific antibodies diluted in 4% BSA in TBS. The following antibodies were used for Western blotting: Ms mAb to BRG1 (Millipore, MABE 121, 1:2000 dilution); Rb mAb to CTCF (Cell Signaling Technology, 3418, 1:1000 dilution); Rb pAb to INI1 (Bethyl, A301-087, 1:5000 dilution).

## Results and Discussion

Prior to sonication we extracted the cross-linked cells twice using extraction and lysis buffer (ELB) as described in Materials and Methods section. The main purpose of this extraction was to separate proteins that are covalently cross-linked to genomic DNA, either directly or through a chain of mediators, from those that are not. This procedure provides enrichment for the fraction of interest, as the ultimate readout of a ChIP experiment is the abundance of DNA co-precipitated by protein pull-down. The ELB extraction protocol described here works well for HeLa, U2OS and IMR90 human cell lines. Some other cell types (e.g. HCT116) may dissolve completely during the extraction, preventing the use of the nuclear envelope for chromatin encapsulation purposes. As intact nuclear envelopes are clearly visible, this process can be monitored by phase-contrast microscopy. In the case of SDS sensitive cells, we recommend titrating down the SDS concentration in the extraction buffer, replacing SDS with Triton X100, or omitting the extraction step altogether.

After extraction, the nuclear pellet was resuspended in a mixture of ELB and water to obtain the final buffer composition of 1:4 ELB:water, assuming that the volume of pellet contributes to the total volume of ELB. In this 4 times diluted ELB, a reduced SDS concentration of 0.1% has two main advantages: this SDS concentration does not precipitate at +4°C (if NaCl concentration is 10 mM or less) and can also be quenched by adding Triton X100 without further sample dilution (final concentrations of 0.1% SDS and 1.0% Triton X100 are compatible with immunoprecipitation by most antibodies). The suspension of extracted nuclei was then solubilized by ultrasound treatment.

In this study, we used a Bioruptor XL water bath sonicator (Diagenode) housed in a cold room (+5…+6°C) that provides both cooling and noise insulation during sonication. To ensure consistency in the water bath temperature, we added 300–400 mL of crushed ice to the water bath 10–15 min prior to sonication. Just before sonicating the samples we carefully removed all of the remaining ice using a sieve, as we found that floating ice is one of the reasons for inconsistency between different sonication experiments, particularly during short ultrasound treatments. During extended sonication all samples eventually reach a plateau of chromatin shearing with a range of 200–400 bp fragments and therefore the effect of ice interference is less apparent in terms of chromatin fragmentation. However, floating ice may contribute to the variability in protein degradation between samples (see below) and therefore we remove ice from the water bath regardless of the sonication time. We determined that even after 20 min of sonication at maximum power (H-setting) in the cold room, the temperature of the water bath increased only to +23°C. Thus, additional water bath cooling with floating ice is usually not required, as under the optimized conditions cell lysis and chromatin fragmentation are accomplished within this timeframe.

To optimize sonication parameters, we first investigated the impact of sample volume on sonication efficiency. One full rotation of the carousel tube holder in the Bioruptor XL takes 24 sec. Therefore we initially reasoned that the sonication cycle should be set at 24 sec ON, 24 sec OFF (not 30 sec ON, 30 sec OFF as recommended in the Diagenode manual v2.1). This is required to ensure that during every sonication cycle each tube is exposed to all intensity zones regardless of its position in the carousel (Fig 1A), and also to prevent phase drift between the cycles (see below regarding positional variation of sonication intensity). Sonication efficiency was assessed by visual inspection of the sample by phase-contrast microscopy, to determine the extent of cells lysis. In contrast to the manufacturer-recommended 200 μL sample volume (Diagenode manual v2.1), we found that sonication of samples as 500 μL aliquots in standard 1.5 mL polypropylene Eppendorf tubes resulted in much faster cell lysis compared to aliquots of higher or lower volumes, titrated within a range of 100–700 μL (Fig 1B).

We asked whether sonication efficiency is equal between different positions of the sonicator in the rotating carousel. Sonication efficiency was determined by agarose gel electrophoresis of purified DNA. This confirmed that in the rotating carousel, sonication efficiency is approximately equal for all positions within the carousel (Fig 1C). This suggests that the observed correlation between sample volume and sonication efficiency is not due to different starting positions of the samples during the experiment but rather a direct consequence of sample volume as all other parameters were kept identical.

Next, we asked if sonication efficiency is equal between different positions in the non-rotating carousel. We inactivated the rotor and aligned the carousels as shown in Fig 1A. Then, we compared sonication efficiency between positions R10, R11, R12, R1, R2, R3 and R4 using low (L) and high (H) power outputs. The results show that not all positions in the water bath deliver sonication energy with equal efficiency and the magnitude of this difference depends on the power setting (Fig 1D). Sonication in position R1 results in the most efficient cell lysis, which is very similar at both power settings. Outside of this “hot spot” sonication efficiency is reduced rapidly for the L-power and slowly for the H-power settings. These results suggest that in a rotating unit, sample sonication occurs primarily as tubes periodically rotate through a limited number of water bath positions.

**Fig 2:**
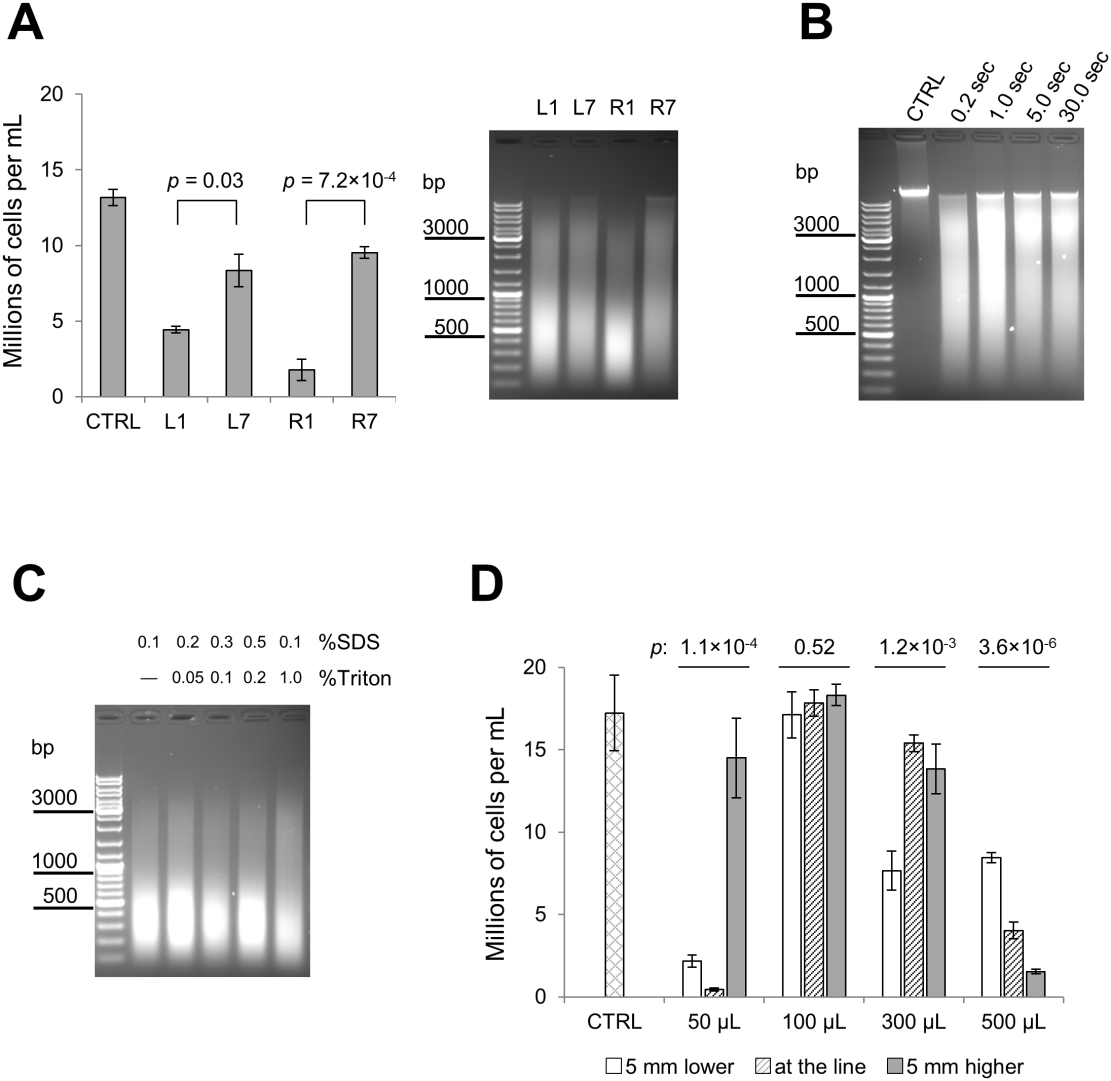
Systematic optimization of sonication conditions. **Part 2. (A)** The effect of sample position on sonication efficiency at low power setting. 500 μL cell suspensions were loaded into positions L1, L7, R1, R7, all other positions were left vacant. Sonication was carried out for 1 min in 1:4 ELB:H_2_O (0.1% SDS final concentration), 5 sec ON/5 sec OFF pulses, no rotation, no ice. Left panel: Intact remaining cells were counted three times and the respective means calculated; error bars reflect the standard deviation. Right panel: Afterwards, the samples were sonicated for an additional 9 min and reverse cross-linked overnight. DNA was purified from the resulting mixture using the Qiagen PCR clean-up kit and analysed on 1.1% agarose gel. The control (CTRL) sample is the cell suspension before sonication. *p*-value for analysis of variance between sonicated samples is 1.0×10^−5^. *p*-values for selected T-tests are shown on the graph. **(B)** The effect of pulse time on sonication efficiency. 500 μL cell suspensions were loaded into position R1; vacant R-positions were filled with tubes containing 500 μL of water. Sonication was carried out for 2 min in 1:4 ELB:H_2_O (0.1% SDS final concentration), with variable pulse times (as indicated), no rotation and no ice. Following sonication, samples were reverse cross-linked overnight. DNA was purified from the resulting mixture using the Qiagen PCR clean-up kit and analysed on 1.1% agarose gel. The control (CTRL) sample is the cell suspension before sonication. **(C)** The effect of buffer composition on sonication efficiency. 500 μL cell suspensions were loaded into position R1; positions R4, R7 and R11 were filled with tubes containing 500 μL of water; other R-positions were left vacant. Sonication was carried out for 10 min in 1:4 ELB:H_2_O supplemented with varying concentrations of SDS and Triton X-100 (as indicated), 5 sec ON/5 sec OFF pulses, no rotation and no ice. Following sonication samples were reverse cross-linked overnight. DNA was purified from the resulting mixture using the Qiagen PCR clean-up kit and analysed on 1.1% agarose gel. **(D)** The effect of water level and sample volume on sonication efficiency. Cell suspensions were loaded into position R1; positions R4, R7 and R11 were filled with tubes containing 500 μL of water; other R-positions were left vacant. Sonication was carried out for 1 min in 1:4 ELB:H_2_O (0.1% SDS final concentration), 5 sec ON/5 sec OFF pulses, no rotation and no ice. Remaining intact cells were counted three times and the respective means calculated; error bars reflect the standard deviation. The control (CTRL) sample is the cell suspension before sonication. Analysis of variance *p*-values are shown on the graph for each sample volume.

Due to the symmetric design of the water bath it would be reasonable to assume that the positions in both carousels are pair-wise equivalents (1–7, 2–6, 3–5, 8–12 and 9–11). However, at least with our instrument, this is not the case. In our tests position R1 performed better than L1, which in turn was more powerful than L7 and R7 (Fig 2A).

After that, using position R1 for the sonication with the rotor inactivated, we studied whether sonication efficiency depends on the length of the ON/OFF cycle. Varying pulse length yielded only a minimal effect (Fig 2B) and therefore for all subsequent experiments with the inactivated rotor we utilized a 5 sec ON/5 sec OFF setting for convenience.

We also tested whether increasing the SDS concentration enhances sonication efficiency, as most protocols suggest 0.5–1.0% SDS in the lysis buffer. For SDS concentrations higher than 0.1% we added Triton X-100 to prevent the SDS from precipitating at low temperature; a minimum amount of Triton X-100 was added in each case. This amount was determined empirically by titrating Triton X-100 concentration in the presence of a fixed concentration of SDS and assessing pellet formation following 1 h incubation on ice. Again, almost no difference was observed between the samples and after only 10 min of sonication at low power the chromatin was sheared to 200–500 bp fragments (Fig 2C).

Finally, we studied the effect of the water level in the water bath on sonication efficiency. Although there is a specific water level recommended for the Bioruptor XL (denoted by a blue line on the inner surface of the bath) it is very difficult to keep this level exactly the same between experiments. This is mostly due to the variable, and user-dependant, angle from which the water meniscus is viewed. To model the potential variation of water level we compared sonication efficiency between the bath filled with water to the line, filled 5 mm below and 5 mm above the line. We sonicated cell suspensions in 50, 100, 300 or 500 μL aliquots. As shown in Fig 2D, water level does affect sonication efficiency, most dramatically in the case of the 50 μL aliquot. This effect might be explained if the hot zone pattern and/or optimal sample volume depends on the water level.

**Fig 3:**
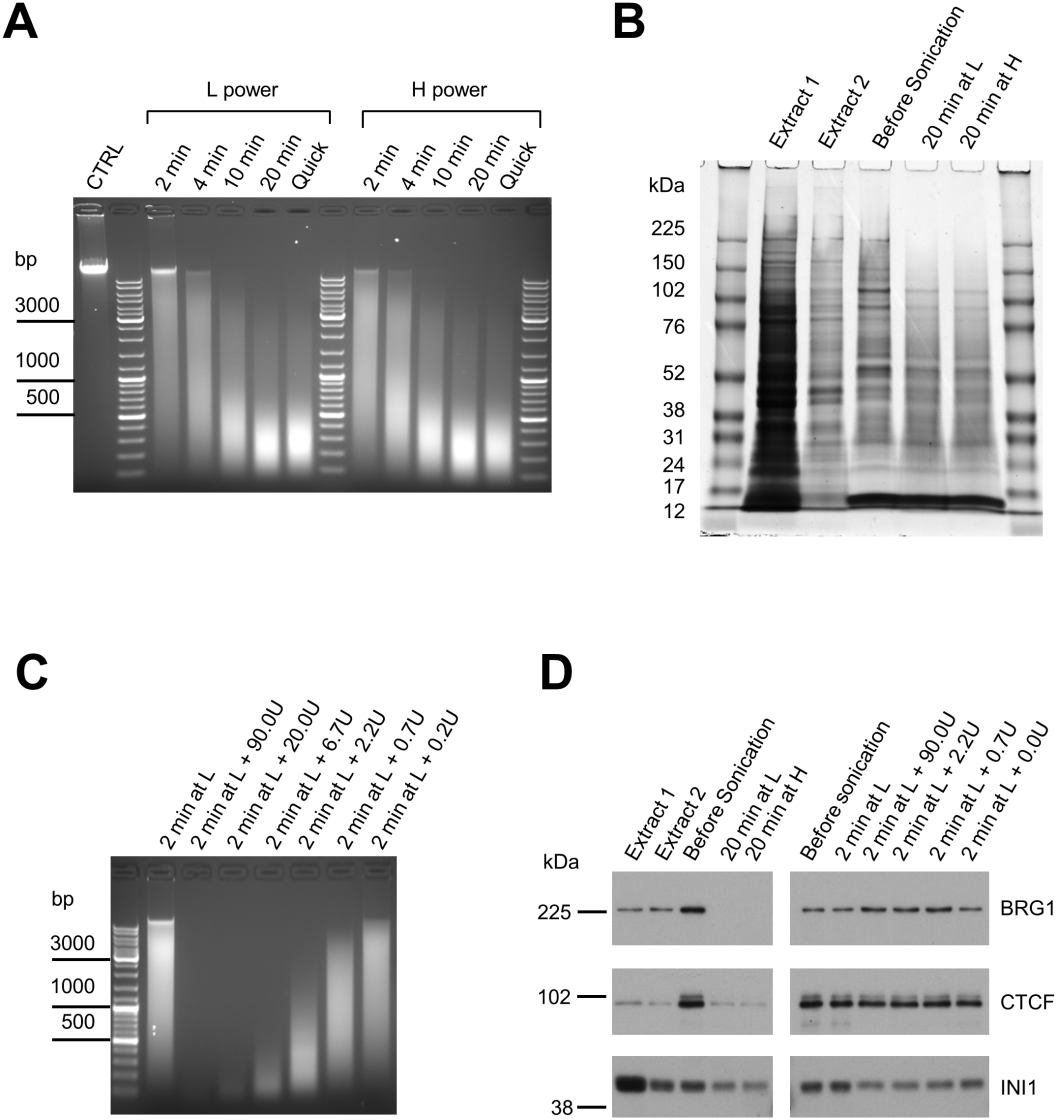
Comparison of chromatin fragmentation by ultrasound alone or in combination with benzonase digestion. **(A)** Comparison of sonication efficiency at the L and H power outputs over time. 500 μL cell suspensions were loaded into position R1; positions R4, R7 and R11 were filled with tubes containing 500 μL of water; other R-positions were left vacant. Sonication was carried out for various times (as indicated) in 1:4 ELB:H_2_O (0.1% SDS final concentration), 5 sec ON/5 sec OFF pulses, no rotation and no ice. Following sonication samples were reverse cross-linked overnight. DNA was purified from the resulting mixture using the Qiagen PCR clean-up kit and analysed on 1.1% agarose gel. The “Quick” lanes contain samples sonicated for 20 min and reverse cross-linked for 1 h at +60°C. **(B)** Coomassie Blue staining of protein fractions generated during the sonication time course shown in panel A. Note the absence of high molecular weight proteins after 20 min of ultrasound treatment. **(C)** Titration of benzonase (0.2U to 90U) to fragment chromatin solubilized by 2 min L-power sonication. Following the digest, samples were reverse cross-linked overnight. DNA was purified from the resulting mixture using the Qiagen PCR clean-up kit and analysed on 1.1% agarose gel. See Material and Methods section for detailed reaction conditions. **(D)** Combination of brief sonication (2 min at L-power) and benzonase digestion (0.7U to 90U) preserves the integrity of large proteins.

Having identified critical sonication parameters, we conducted a 20 min time course at the low (L) and high (H) power outputs using the following parameters: no floating ice; water bath filled to the line and cooled to +4C; 500 μL samples in 0.1% SDS (1:4 ELB:H_2_O) placed in position R1; no rotation; 5 sec ON/5 sec OFF pulses. Both power settings performed equally well, consistently yielding 200–500 bp fragments after 10 min and 150–400 bp after 20 min (Fig 3A). However, the low power setting has the advantage of reduced sample heating compared to the H-setting.

Since sonication can potentially degrade proteins and denature protein epitopes, we next assessed the impact of sonication on protein integrity. To do this, we combined the samples from Fig 3A with 4x Laemmli sample buffer containing DTT (100 mM final concentration), incubated 30 min at +97°C to reverse the cross-links and analysed by SDS-PAGE along with extract supernatants from the same experiment. Fig 3B demonstrates that, as discussed previously, a large proportion of the total protein was removed from the sample during extraction, significantly reducing sample complexity and presumably enriching for chromatin-bound targets. More importantly, we observed a drastic decrease in Coomassie Blue staining efficiency for higher molecular weight proteins when comparing samples before sonication and after 20 min of sonication, at either the L- or H-power settings. Western blotting of 184 kDa BRG1, 83 kDa CTCF and 44 kDa INI1 confirmed this observation (Fig 3D, left panels). Taken together, these results suggest that large proteins, similar to genomic DNA, are prone to degradation by ultrasound treatment.

Degradation of proteins is likely detrimental to efficient chromatin immunoprecipitation. To overcome this limitation, we prepared input chromatin by combining brief sonication and benzonase digestion, to limit the destructive action of the ultrasound. In the first step, the sample was sonicated for the minimal time required to lyse the cells and solubilize the chromatin, i.e. 2 min at L-power. Under these conditions, the DNA was sheared to relatively large fragments (Fig 3C, left lane). Next, the sample was treated with 0.2U to 90U of benzonase for 15 min as described in the Materials and Methods section (Fig 3C). By this protocol, even when DNA was fragmented to subnucleosomal sizes, antigen reactivity by Western blot was preserved (Fig 3D). Hence, this modified approach allows for the generation of various sized chromatin fragments (depending on the amount of benzonase), while preserving the integrity of associated proteins. Replacing bezonase with sequence-specific DNA endonucleases, such as AluI or CviQI four-cutters, may provide additional benefit of preserving RNA-mediated interactions and the convenience of digest saturation albeit at the expense of biasing against genomic loci that lack the corresponding consensus.

To summarize, we systematically characterized the performance of the Bioruptor XL sonicator (Diagenode) and the described approach should be generally applicable to the rational optimization of any water bath sonicator. With respect to the Bioruptor XL, we observed that the sonication efficiency strongly depends on the sample volume, and concluded that 500 μL of sample per 1.5 mL tube is the optimal volume. In the non-rotating carousel, sonication efficiency is greatly affected by the sample position within the water bath, with distinct ‘hot’ and ‘cold’ zones present. Despite the symmetric design of the water bath, we were unable to identify two equally efficient sonication positions. Therefore, when processing multiple sample tubes, we recommend using the rotating carousel with 24 sec ON/24 sec OFF pulses, the high power setting, an ice-cold water bath filled to the line and no floating ice in order to ensure that all samples are treated identically by passing through all intensity zones of the water bath. The total treatment time required may be relatively long (20–30 min), as each sample will spend only a fraction of the total time in the hot zones where cell lysis and chromatin fragmentation occur. When the experiment is limited to 1 or 2 sample tubes we recommend sonicating samples in position R1 with no rotation, 5 sec ON/5 sec OFF pulses, the low power setting, and an ice-cold water bath with no floating ice. We consider a 1:4 ELB:H_2_O mixture to be the optimal sonication buffer, since increasing SDS concentration above 0.1% does not enhance chromatin fragmentation but does require higher sample dilution post-sonication, which may reduce immunoprecipitation efficiency.

High temperature treatment is used to reverse formaldehyde cross-linking and dissociate DNA-protein and protein-protein complexes into individual components. When the component of interest is DNA the treatment is performed at 60°C and high salt concentration to prevent dissociation of DNA strands. This is typically accomplished by an overnight incubation of at least 8 hours. Here we demonstrate that in the presence of Proteinase K, fragmentation of DNA can be evaluated already after 1 hour treatment because extending the procedure to overnight makes almost no difference in terms of agarose gel analysis (Fig 3A, “20 min” lane versus “Quick” lane). Therefore, we recommend using the quick reverse cross-linking method to enable prompt evaluation of chromatin quality as a checkpoint to decide whether to proceed with immunoprecipitation or to perform additional sonication. DNA purification for more challenging types of downstream analysis, such as ChIP-seq, should be performed using standard overnight protocol to ensure maximum possible reversal of cross-links.

We have also demonstrated that ultrasonic fragmentation of chromatin to sizes below 400 bp (typically required for ChIP-seq applications) results in degradation of high molecular weight proteins, and probably large protein complexes. In contrast, it is likely that histones, due to their low molecular weight, tight packaging within the nucleosome and direct contact with DNA, are only minimally degraded by ultrasound treatment, even during extended sonication. Consequently, experiments involving the ChIP of histones are less likely to benefit from utilisation of the benzonase method described above. However, the combination of minimal sonication and benzonase digestion should greatly improve chromatin immunoprecipitation of high molecular weight proteins, such as the chromatin remodeller BRG1 and insulator protein CTCF, by preserving the corresponding antigens and complex protein assemblies cross-linked to DNA.
